# Governmental Restrictions and Real Estate Investor Risk Perception

**DOI:** 10.1007/s11146-023-09947-y

**Published:** 2023-03-27

**Authors:** Carina Kaiser, Julia Freybote, Wolfgang Schäfers

**Affiliations:** 1 International Real Estate Business School (IRE|BS), University of Regensburg, Universitaetsstr. 31, 93053 Regensburg, Germany; 2 School of Business, Portland State University, 615 SW Harrison St, Portland, OR 97201 USA

**Keywords:** Commercial Real Estate Investment, Risk Perception, Government Risk, COVID, Political Attitudes

## Abstract

We investigate the impact of governmental restrictions on the short-term risk perception, as proxied by the going-in cap rate, of investors in regional and neighborhood shopping centers. We use the COVID-19 pandemic as a natural experiment and proxy for the length and severity of COVID-19 restrictions with the political affiliation of state governors. Using a sample of 40 metropolitan statistical areas (MSAs) across 27 states over the period of 2018 to 2021, we find that for states with Republican governors, which proxy for shorter and fewer COVID-19 restrictions, investors in regional malls required a lower going-in cap rate in the pandemic period than for states with Democratic governors. This effect does not exist for neighborhood shopping centers, whose tenants were not as affected by COVID-19 restrictions. Robustness checks suggest that our findings can be explained with mask mandates as one type of governmental restrictions, and that COVID-19 related restrictions do not impact the long-term risk perception of retail real estate investors. We furthermore find that the political attitudes of an MSA have an impact on investor risk perception.

## Background

Characteristics of geographical real estate markets such as economic growth (Feng & Wu, 2021) or location density (Fisher et al., 2022) impact the investment risk for investors. Local political decision-making represents another risk factor. Political uncertainty has been found to affect residential property values (Monfared & Pavlov, 2019), commercial risk premiums (Chau, 1997) as well as construction activity (Luo et al., 2021). Governmental policies targeting multifamily, such as rent control or inclusionary zoning, have been shown to influence investor behavior in terms of development, tenant screening, or divestment from the affected market (Ambrose & Diop, 2021; Asquith, 2019; Diamond et al., 2019a, b; Schuetz et al., 2011; Suzuki & Asami, 2020).

Previous studies on the impact of political decision-making on real estate markets have focused on single- and multifamily housing and ignored other commercial property types. The purpose of this study is to investigate the impact of governmental restrictions on the risk perception of commercial real estate investors. Hereby, we define investor risk perception as their beliefs about the possibility of loss, which is reflected in return requirements.

One challenge of investigating governmental restrictions in the context of commercial real estate markets is the difficulty of capturing them at the local level. Fortunately, the COVID-19 pandemic represents a unique opportunity for our empirical investigation. As COVID-19 cases increased across US states, Republicans and Democrats increasingly viewed the outbreak from different angles, ranging from personal health risks to prioritizing the conveniences of everyday life (Pew Research Center, 2021). As the federal government did not take the lead in proposing nationwide policies, responses to the pandemic such as social distancing, mask mandates, or stay at home orders were left to state governments. The result was a clear distinction along party lines on actions taken with Democratic-led states imposing stricter and longer public health measures than Republican-led states. The political polarization also resulted in a partisan split in the risk perception of the disease (Benton et al., 2021).

In our analysis, we measure local governmental restrictions with the political affiliation of the state governor. Hereby, Democratic governors proxy for more severe and longer COVID-19 restrictions than Republican governors. This is consistent with recent studies showing that governor partisanship is the most important explanation for differences in social distancing policies and mask mandates across states during the pandemic (Adolph et al., 2021, 2022).

We employ a quasi-experimental design to analyze the effects of governmental restrictions, proxied by the political affiliation of governors, on the risk perception of real estate investors in the pre-COVID (2018/2019) and COVID (2020/2021) period. We proxy for the risk perceived by investors using the survey-based Situs RERC going-in cap rate, which captures a short-term ex-ante risk premium. While local governmental restrictions during the COVID-19 crisis had an impact on several commercial property types, such as office (e.g., stay at home orders) and multifamily (e.g., eviction moratoriums), we focus on retail real estate, particularly neighborhood and regional shopping centers. Previous studies find a particularly strong impact of the pandemic on this property type (Hoesli & Malle, 2021; Ling et al., 2020; Milcheva, 2022; van Dijk et al., 2020). The strength of this impact, however, varied across shopping center types and retail segments. While regional shopping centers with tenants focused on leisure shopping, food, and non-essential goods were impacted by regulations such as mask mandates, social distancing, and limits on store occupancy, which resulted in consumers shifting to online shopping, neighborhood shopping centers with tenants focused on essential goods such as groceries were less affected by governmental restrictions.

We hypothesize that stricter and longer governmental restrictions imposed by Democratic governors during the pandemic led to a higher short-term risk perception of retail real estate investors and thus a higher required going-in cap rate for shopping centers in Democratic-led states. We also expect this effect to be stronger for regional malls than neighborhood shopping centers, due to the nature of tenants and shopper attitudes.

Using a sample of 40 metropolitan statistical areas (MSAs) across 27 states over the period of 2018 to 2021, we firstly find that the political affiliation of a state governor impacts investor risk perception. Irrespective of the shopping center type, investors require a higher going-in cap rate in Republican-led states than Democratic-led states. Secondly, the COVID-19 pandemic, unsurprisingly, increased the risk perception of retail real estate investors. However, during this period, the political affiliation of the state governor, proxying for the length and severity of restrictions, impacted the investor risk perception for regional malls. In particular, retail real estate investors required a lower going-in cap rate for regional malls in MSAs located in Republican-led states. This effect does not exist for neighborhood shopping centers. In a robustness check, we show that mask mandates as one type of governmental restrictions represent an explanation for our results. Furthermore, we find no impact of COVID-19 related governmental restrictions on the long-term risk perception of retail real estate investors, as proxied by the required ex-ante pre-tax yield (IRR). We also show that the political attitudes of an MSA’s population have an impact on investor risk perception. Compared to Democratic-leaning MSAs, investors require a higher going-in cap rate for Republican-leaning MSAs, irrespective of mall type. One explanation is that the political attitudes of an MSA proxy for factors such as population growth, income, or diversity that impact future space and asset market conditions.

To our knowledge, this is the first study to investigate the impact of government restrictions on the risk perception of commercial real estate investors. In the context of real estate, risk perception has been primarily studied in housing and residential mortgage markets, particularly with regards to environmental risks (Duanmu et al., 2022; Liao et al., 2022; Pollack & Kaufmann, 2022; Xu & Xu, 2020; Yi & Choi, 2020). The risk perception of commercial real estate investors has been neglected in the literature with a few exceptions such as Beracha et al. (2019) and Chau (1997). We contribute to the literature on the impact of governmental restrictions on real estate market participants (Ambrose & Diop, 2021; Asquith, 2019; Diamond et al., 2019a, b; Luo et al., 2021; Monfared & Pavlov, 2019; Suzuki & Asami, 2020) by (1) focusing on a non-housing property type and (2) focusing on investor risk perception.

We furthermore contribute to an emerging stream of literature investigating the impact of the COVID-19 pandemic on commercial real estate markets (Wang & Zhou, 2022; Hoesli & Malle, 2021; Ling et al., 2020; Milcheva, 2022; van Dijk et al., 2020), which is part of a larger real estate literature on the effects of the pandemic as an exogenous shock on, amongst others, housing, mortgage, and REIT markets (Anderson et al., 2022; Milcheva, 2022; Pence, 2022; Zhang et al., 2022; Zhou et al., 2022; D’Lima et al., 2021).

The remainder of this paper is structured as follows. Next, we review the relevant literature, which is followed by a discussion of our data and methodology. Then, we present our results and a conclusion.

### Literature Review

Previous studies in the finance and real estate literature provide evidence for the impact of political uncertainty and governmental regulations on corporate and real estate investments. Political uncertainty has been found to impact corporate investments (Azzimonti, 2018; Çolak et al., 2017; Gulen & Ion, 2015; Jens, 2017; Julio & Yook, 2012; Nguyen & Phan, 2017). In particular, M&A activities and corporate investments have a negative relation with political uncertainty. Studies focused on corporate financing activities find that an increase in political uncertainty is associated with higher debt financing costs (Francis et al., 2014; Waisman et al., 2015) and declining equity financing (Chan et al., 2021; Çolak et al., 2017).

A number of studies investigate political uncertainty in the context of housing markets. Bahmani-Oskooee and Ghodsi (2017) and Choudhry (2020) examine the impact of economic policy uncertainty on house prices in the US and England. Both studies find predominantly negative effects. Another set of studies supports that homeowners tend to vote in favor of safeguarding or increasing real estate values (Brunner et al., 2001; Brunner & Sonstelie, 2003; Dehring et al., 2008; Zahirovic-Herbert & Turnbull, 2009; Luo et al., 2021) find that building permits on US state-level are negatively associated with aggregate political uncertainty. Monfared and Pavlov (2019) study the impact of the Brexit referendum on residential real estate prices in areas of London and find that areas with a higher concentration of EU passport holders or highly educated residents experienced a disproportionately large price decline following the vote.

Another stream of literature focuses on governmental policies in the context of single-family housing markets such as property taxes (Hoyt et al., 2011), conservation districts (Diaz et al., 2008), anti-discrimination (Bostic & Martin, 2005), loan-to-value restrictions (Armstrong et al., 2019) or land use regulations (Lima & Silveira Neto, 2019; Takeda et al., 2019).

While political uncertainty and governmental regulations have received considerable attention in the housing literature, they have received less attention in the commercial real estate literature. Previous studies primarily focus on the multifamily market and governmental regulations such as inclusionary zoning (Schuetz et al., 2011), rent control (Asquith, 2019; Diamond et al., 2019a, b), evictions (Suzuki & Asami, 2020), and other regulations (Ambrose & Diop, 2021). Overall, these previous studies suggest that multifamily investors respond to governmental restrictions by, amongst others, shifting their investment strategies, changing their supply of space, or increasing their tenant screening. Chau (1997) is the first study to investigate political uncertainty in the context of non-residential commercial real estate markets. In particular, the author investigates the impact of the 1997 repossession of Hong Kong by China on risk premiums for residential and commercial real estate and provides evidence of an increase in risk premiums for commercial property types.

In addition to governmental regulations and political uncertainty, the political attitudes of managers have been found to impact corporate decision-making. Hutton et al. (2014) find that the political leaning of corporate executives impacts investment, financing, and other corporate decisions. Subrahmanyam et al. (2020) show that CEO characteristics impact political donations and that firms have a preference to donate to local politicians in the state a company is headquartered, emphasizing the importance of local politics to firms. Focusing on the COVID-19 pandemic, Benton et al. (2021) find that the political leaning of a firm’s management impacts the voluntary disclosure of COVID-19 risks and political donations. One explanation for these findings is that Democratic-leaning managers perceived the disease’s risks to be higher than Republican-leaning.

Several studies provide evidence that the political orientation of CEOs affects corporate social responsibility (CSR). Chin et al. (2013) provide evidence that the liberalism or conservatism of CEOs impact CSR engagement. Di Giuli and Kostovetsky (2014) find that the political leaning of founders, CEOs, directors as well as the headquarters’ states determine CSR engagement. Jeong and Kim (2020) show that CEO liberalism positively predicts CSR, albeit results vary depending on the political affiliation of the US president. Gupta et al. (2021) find that the political leaning of CEOs impacts the adoption of CSR executives. Focusing on REITs, (Deng et al., 2021) show that the political leaning of CEOs of REITs affects business decisions. In particular, Democratic-leaning CEOs are willing to take on more risks, e.g., in terms of financing decisions, and adopt more (environmental) ESG strategies than Republican-leaning CEOs.

Early studies analyzing the effects of the COVID-19 pandemic on commercial real estate markets show that retail real estate markets were the most affected, which motivates the focus of our study on this property type. Van Dijk et al. (2020) use the liquidity impact at the beginning of the pandemic to forecast future price changes in commercial real estate markets. They project that retail real estate markets will be most affected, with price declines in the range of 14–19%. Focusing mostly on European real estate markets, Hoesli and Malle (2021) analyze the effects of the COVID-19 pandemic on commercial real estate prices. They find property prices in the retail and hospitality sector to be affected to the highest degree. Ling et al. (2020) examine US REIT returns in the context of the geographical exposure of REIT portfolios to COVID-19. They find a negative reaction of returns to increasing case numbers. The most negative response across all REIT property types is found for firms focused on retail and residential real estate, while those focused on healthcare are positively correlated with COVID-19 growth. Milcheva (2022) shows that the effect of COVID-19 is associated with steep declines in international real estate security returns and an increase in risk. Retail real estate is found to have the highest sensitivity to COVID-19, whereas healthcare has the lowest sensitivity. Generally, in line with Ling et al. (2020), she finds that US REITs perform significantly worse the higher their exposure is to COVID-19.

We assume that longer and stricter governmental restrictions in response to the COVID-19 pandemic increase the cash flow uncertainty for retail real estate investors and thus their perceived investment risk in the short-term. As a result, investors are expected to require a higher risk premium, as reflected in higher going-in cap rates, for shopping centers in Democratic-led states during the pandemic. This is consistent with recent studies showing that governor partisanship is the most important explanation for differences in social distancing policies and mask mandates across states during the pandemic (Adolph et al., 2021, 2022). However, considering that neighborhood shopping centers were less affected by restrictions due to the essential nature of their tenants, we expect the impact of governmental restrictions on investor risk perception to be lower for this type of shopping center compared to regional malls with their tenant mix aimed at dining out, entertainment, and leisure shopping.

### Data and Methodology

To measure the short-term risk perception of retail real estate investors in regional malls and neighborhood centers, we obtain the going-in cap rate (*GICAPR*) for the respective mall types from Situs RERC. This cap rate represents an ex-ante return required by investors responding to the Situs RERC survey based on current risk perception and market information. We hereby follow Beracha et al. (2019), who use the survey-based Situs RERC data as basis for their ex-ante return measure. Please note that one limitation of our study is that we do not have information on the political orientation of market participants responding to the Situs RERC survey. The political attitudes of individual investors likely impact their risk perception, particularly with regard to the COVID-19 pandemic. Future studies with the appropriate datasets may further investigate the impact of political attitudes of real estate investors on their risk perception.

While Situs RERC survey data is not derived from transactions and relies on the voluntary participation of real estate market participants, we assume that the data reflects unbiased estimates of investors’ expectations. Clayton et al. (2009) compare Situs RERC capitalization rates with those derived from real estate transactions (Real Capital Analytics and National Council of Real Estate Investment Fiduciaries). The authors find that all three cap rate series are in near perfect agreement, providing reasonable assurance that the RERC survey data is reflective of market behavior. Situs RERC going-in cap rates are available for 40 MSAs across 27 states. We obtain the data from Situs RERC for the period of the first quarter of 2018 to the fourth quarter of 2021. The years 2018/19 indicate the pre-pandemic control (base) period and 2020/2021 represent the COVID-period.

In our empirical investigation, we take advantage of the sharp divide in attitudes and responses to the COVID-19 pandemic between the Democratic and Republican party. Depending on political leaning, the US population has been divided on many pandemic-related issues. Some of these directly affect retail real estate. For example, Republicans were far more comfortable than Democrats going to hair salons (72% vs. 37%), restaurants (65% vs. 28%), or indoor events (40% vs. 11%; Pew Research Center, 2020).

Our independent variable of interest is a state governor’s party affiliation for the respective MSA in our sample, coded 1 for a Republican governor and 0 for a Democratic one (*REPUBGOV*). This proxy is suitable, as governors are responsible for implementing state laws and monitoring the work of the state executive branch. With that, they promote and track new and amended policies and programs through a variety of tools (e.g., executive orders, executive budgets, legislative proposals and vetoes), and thus, were responsible for pandemic-related restrictions.^1^ Recent studies show that governor partisanship, rather than health or economic conditions, is the most important explanation for differences in pandemic-related restrictions across states (Adolph et al., 2021, 2022). We create a binary variable for the COVID-19 pandemic (*COVID*) which equals to 1 for 2020/21 and 0 for 2018/19. Then, we create interaction effects of *COVID* and *REPUBGOV* to capture the pandemic-specific impact of governmental restrictions on investor risk perception.

We control for several variables that affect going-in cap rates. At MSA-level, we first obtain the population (*POPUL*) as well as the annual population growth rate (*POPULGrowth*) from the US Census Bureau (Federal Reserve Economic Data). We also include the quarterly unemployment rate (*UNEMPL*) for each MSA, obtained from the US Bureau of Labor Statistics, and the per capita income (*PCI*) of an MSA from the US Bureau of Economic Analysis. Frey (2021) shows that, based on the 2020 census, the majority of the largest MSAs grew faster than in the past and became more racially diverse. Thus, diversity of an MSA proxies for future population growth, which is an important demand driver for retail real estate. Furthermore, research has shown that diversity plays an important role in reducing poverty, expanding opportunity, and promoting economic mobility (Chetty et al., 2014; Chetty & Hendren; Cortright, 2018; Zhang & Logan, 2016). Based on Frey (2021), we create a dummy variable indicating if an MSA classifies as race-ethnically diverse (*DIVERSE*). *DIVERSE* is coded 1 if the percentage of the white population is below the average of all MSAs in the sample (54.21%) and 0 if it exceeds the average.

We also capture market conditions in retail real estate space and asset markets by including quarterly property type-specific information obtained from the Situs RERC survey. In particular, we include leasing assumptions with regard to the renewal probability of tenants in percent (*RENPROB*), the marketing time in months (*MARKT*), and the assessment of investment conditions (*INVCOND*) on a scale of one (poor) to ten (excellent).

Summary statistics are presented in Table 1. The party affiliation of state governors is balanced between Democratic and Republican (50% each). The average MSA size is just over 4.1 million people. However, the standard deviation is comparably large, indicating wide gaps between populations. On average, the number of people living in an MSA increased by 0.71% per year during the sample period. About 47% of MSAs in our sample can be considered diverse, considering the percentage of the white population accounts for less than 54.21%.

**Table 1:** Descriptive Statistics

	Mean	Median	Std. Dev.	Max	Min
**REPUBGOV**	0.50	0.00	0.50	1.00	0.00
**DIVERSE**	0.47	0.00	0.49	1.00	0.00
**POPUL**	4129.48	2805.80	3498.43	20096.41	1204.75
**POPULGrowth**	0.71	0.69	1.02	4.49	-2.45
**UNEMPL**	4.89	4.10	2.52	18.50	1.20
**PCI**	63445.52	60911.18	14743.18	129889.1	38,418
*Regional Mall*					
** GICAPR**	7.01	7.00	0.52	8.60	6.00
** RENPROB**	62.02	61.80	1.61	65.80	59.70
** MARKT**	9.45	10.00	1.27	11.10	7.40
** INVCOND**	3.25	3.00	0.83	4.70	2.20
*Neighborhood Center*					
** GICAPR**	6.80	6.80	0.45	8.20	5.80
** RENPROB**	67.38	67.05	1.97	71.40	64.50
** MARKT**	6.95	7.22	0.78	8.40	6.00
** INVCOND**	5.45	5.31	0.54	6.10	4.30

Note: This table presents the descriptive statistics aggregated by MSAs (N = 40) and where applicable, disaggregated at property type level. The sample period spans Q1/2018 to Q4/20 2021. REPUBGOV is the state governor’s political party and is coded 1 for republican; 0 for democratic. DIVERSE is based on the race-ethnic composition of an MSA and coded 1 if the percentage of the white population is below the average of all MSAs in the sample; 0 if it exceeds the average. POPUL is the resident population estimate in thousands of persons, POPULGrowth its percent change from a year ago. UNEMPL is the civilian unemployment rate. PCI is the first difference of the per capita income level. Based on the Situs RERC survey, GICAPR is the going in cap rate, RENPROB is the renewal probability, MARKT is the marketing time in month, and INVCOND is investment conditions rated on a scale of 1 = poor to 10 = excellent.

Going-in cap rates are slightly higher for regional malls than for neighborhood centers reflecting the higher risk of this shopping center type to investors due to, e.g., a higher ecommerce and discount competition (Kaiser & Freybote, 2021). Other property market variables provide further evidence for the lower risk of neighborhood centers compared to regional malls. Investment conditions and tenant renewal probability are higher, on average, for neighborhood centers (5.45; 67%) than for regional malls (3.25; 62%). While marketing time for regional malls is 9.45 months on average, it is just under 7 months for neighborhood centers.

We assess the stationarity of variables using the Im-Pesaran-Shin test for MSA-specific variables (*GICAPR, POPUL, POPULGrowth, UNEMPL, PCI*) as this test assumes panel-specific autoregressive parameters and heterogeneous variances across panels. For variables that do not vary across panels, i.e., have the same autoregressive parameter (*RENPROB, MARKT, INVCOND*), the Breitung test was applied. After integrating *PCI* at first order, the results for all variables suggest that the null hypothesis of (all) panels containing unit roots can be rejected.

We conduct a number of diagnostic tests to assess heteroskedasticity, serial and contemporaneous correlation. First, we conduct the Pesaran and Friedman cross-sectional dependence tests to assess whether residuals are cross-sectionally correlated (Hoechle, 2007; Hoyos & Sarafidis, 2006). Both tests suggest the presence of contemporaneous correlation in our dataset. Next, we conduct the Wooldridge test for serial correlation in panel data, which indicates serial correlation. Last, the Wald test suggests the presence of heteroskedasticity in our panels.

To control for heteroskedasticity, contemporaneous and serial correlation, we estimate the model in Eq. (1) using a panel regression with correlated panel-corrected standard errors (PCSE). Our panel variable is the MSA ID and the time variable is the quarter & year. The autocorrelation is assumed to be panel-specific (AR(1)) and computed based on the autocorrelation of residuals. Errors are also set to be panel-level heteroskedastic and correlated across panels. The regression with PCSE is preferable to a GLS model controlling for auto- and cross-sectional correlation, as the latter is inappropriate for the present data set given that the number of groups (N) is larger than the time periods (T) (Hoechle, 2007). The model can be written as

1 $${ Y}_{it}={\beta }_{1}{X}_{it}+{\beta }_{2}{Z}_{it}+{ \alpha }_{i}+{\epsilon }_{it}$$

where ${ Y}_{it}$is the mall type-specific going-in cap rate (*GICAP*) for MSA *i* in quarter *t*, ${X}_{it}$ represents our independent variables of interest (*REPUBGOV, COVID*, and the interaction term), and ${Z}_{it}$ is a vector of our control variables (*POPUL, POPULGrowth, UNEMPL, RENPROB, MARKT, INVCOND* and *PCI*). ${ \alpha }_{i}$ represents MSA-specific effects, and ${\epsilon }_{it}$ is the idiosyncratic error.

## Results

Table 2 presents the results for the going-in cap rate (*GICAPR)* based on Eq. (1) with Model 1 showing the results for regional malls and Model 2 for neighborhood centers. The coefficients on *COVID* are highly significant and positive for both shopping center types, indicating the increase in investor risk perception due to the higher pandemic-induced cash flow uncertainty. Compared to the pre-COVID period, retail real estate investors require an additional risk premium to going-in cap rates in the COVID period.

**Table 2:** Results for Going-In Cap Rate and State Governor

	Model 1		Model 2	
	Regional Mall		Neighborhood Center	
	Coef.	SE	Coef.	SE
**COVID**	0.36***	**0.07**	0.24***	**0.06**
**REPUBGOV**	0.07**	**0.03**	0.08**	**0.04**
**REPUBGOV#COVID**	-0.07***	**0.03**	0.03	0.03
**UNEMPL**	-0.01**	**0.006**	-0.01**	**0.005**
**RENPROB**	-0.02*	**0.01**	-0.01**	**0.006**
**MARKT**	0.01	0.02	0.03	0.03
**INVCOND**	0.06*	**0.03**	0.07**	**0.03**
**DIVERSE**	-0.13***	**0.04**	-0.21***	**0.03**
**POPUL**	< 0.0001	< 0.0001	0.00001**	**< 0.0001**
**POPULGrowth**	-0.02***	**0.01**	-0.01	0.01
**PCI**	-0.00001**	**< 0.0001**	< 0.0001	< 0.0001
**Constant**	7.79***	**0.75**	6.95***	**0.44**
*N*	*600*		*600*	
*No of groups*	*40*		*40*	
*Avg. obs*	*15*		*15*	
*Wald χ* ^*2*^	79.95***		110.43***	

Note: This table presents the results for the regression (panel-specific AR, autocorrelation is calculated based on the autocorrelation of residuals, heteroskedastic panels) for GICAPR. All variables are as defined in Table 1.

*, **, and *** denote statistical significance at the 10, 5, and 1% level respectively.

The positively significant coefficients on *REPUBGOV* (main effect) for both mall types indicate that the party affiliation of a state’s governor affects going-in cap rates. In particular, compared to Democratic-led states, going-in cap rates for both mall types are significantly higher in Republican-led states. We will further investigate this finding in the remainder of this study.

The interaction effect of *REPUBGOV* and *COVID* is significantly negative for regional malls. Thus, compared to Democratic-led states, investors require a lower going-in cap rate for regional malls in Republican-led states during the pandemic. This suggests that the political affiliation of a governor, proxying for the lengths and severity of COVID-19 restrictions, impacts investor risk perception in terms of going-in cap rates during the pandemic. As the main effect of *REPUBGOV* is positive and the interaction effect of *REPUBGOV* and *COVID* is negative, our results suggest that during the pandemic, the risk premium required by regional mall investors is reduced for Republican-led states. Or put differently, the COVID pandemic moderates the relation of state governor affiliation and going-in cap rates for regional malls.

On the other hand, the results for Model 2 indicate no impact of governmental restrictions on the risk perception of investors in the COVID period for neighborhood centers. The essential nature of their tenants protected these types of shopping centers from imposed temporary store closures during the pandemic, and at the same time customers continued to visit, for example, grocery stores despite health risks or the inconvenience of lines or mask mandates. The results for the interaction effect of *REPUBGOV* and *COVID* for regional and neighborhood shopping centers are in line with our expectations.

It is also worth noting that *DIVERSE* has a highly significant negative relation with going-in cap rates in both models, suggesting that an ethnical-diverse population composition in an MSA presents a lower risk to retail real estate investors. This is consistent with economic research, which shows that diversity can have an impact on, among others, economic mobility and thus, economic potential (Chetty et al., 2014; Chetty & Hendren; Cortright, 2018; Zhang & Logan, 2016).

To assess whether our findings in Table 2 are indeed driven by COVID-19 related restrictions, we next examine the relation between mask mandates and going-in cap rates. We focus on mask mandates as a type of governmental restrictions for several reasons. First, the respective data on mask mandates is available on a monthly basis for the sample period and all MSAs. Quarterly or monthly data for other COVID restrictions such as in-store occupancy limits or social distancing requirements were not available for all MSAs in our sample. Second, mask mandates have particularly influenced public opinion. Adolph et al. (2022) find the presence of a Republican governor to be the most important predictor for the delay of indoor mask mandates.

Using data from the Centers for Disease Control and Prevention, we create a dummy (*MASKM*) equaling 1 if a public mask mandate was in place on the majority of days in a quarter and 0 if it was not. As the name implies, a public mask mandate requires facemasks to be worn in public. More specifically, individuals operating in a private capacity are required to wear a mask anywhere outside their homes, including retail businesses and restaurants. We estimate a modified model with *MASKM* as our independent variable of interest (Model A) and *MASKM* and its lag to capture whether mask mandates continued from the previous quarter (Model B).

The results are presented in Table 3. For regional malls, the coefficient on *MASKM* in Model A is significantly positive, albeit only at the 10% level. If we include the lag of *MASKM* (*L.MASKM*; Model B), the coefficient on *MASKM* becomes insignificant and the coefficient on *L.MASKM* is significantly positive at the 5% level. These results suggest that mask mandates lead to a cap rate premium for this mall type, independent from the *COVID* premium. On the other hand, mask mandates have no impact on going-in cap rates for neighborhood centers.

**Table 3:** Robustness Check: Mask Mandate

	Model 1 Regional Mall				Model 2 Neighborhood Center			
	Model A		Model B		Model A		Model B	
	Coef.	SE	Coef.	SE	Coef.	SE	Coef.	SE
**COVID**	0.33***	**0.06**	0.32***	**0.06**	0.26***	**0.05**	0.26***	**0.05**
**MASKM**	0.05*	**0.03**	0.04	0.03	0.002	0.03	0.01	0.03
** L.MASKM**			0.07**	**0.03**			0.01	0.03
**UNEMPL**	-0.02**	**0.01**	-0.01**	**0.006**	-0.01**	**0.005**	-0.01**	**0.005**
**RENPROB**	-0.02*	**0.01**	-0.02**	**0.01**	-0.01**	**0.006**	-0.01**	**0.006**
**MARKT**	0.01	0.02	0.003	0.02	0.03	0.03	0.02	0.03
**INVCOND**	0.06*	**0.03**	0.06*	**0.03**	0.07**	**0.03**	0.08***	**0.03**
**DIVERSE**	-0.14***	**0.04**	-0.16***	**0.03**	-0.21***	**0.03**	-0.20***	**0.03**
**POPUL**	< 0.0001	< 0.0001	< 0.0001	< 0.0001	< 0.0001	< 0.0001	< 0.0001	< 0.0001
**POPULGrowth**	-0.02**	**0.01**	-0.02**	**0.008**	-0.005	0.01	-0.003	0.01
**PCI**	-0.00002***	**< 0.0001**	-0.00002**	**< 0.0001**	< 0.0001	< 0.0001	< 0.0001	< 0.0001
**Constant**	7.84***	**0.73**	7.88***	**0.72**	7.01***	**0.44**	6.99***	**0.45**
*N*	*600*		*600*		*600*		*600*	
*No of groups*	*40*		*40*		*40*		*40*	
*Avg. obs*	*15*		*15*		*15*		*15*	
*Wald χ* ^*2*^	83.01***		100.38***		81.99***		89.83***	

Note: This table presents the results for the regression (panel-specific AR, autocorrelation is calculated based on the autocorrelation of residuals, heteroskedastic panels) for GICAPR. MASKM is 1 if public mask mandate was in place; 0 if not. L.MASKM is the lag of the respective variable. All other variables are as defined in Table 1.

*, **, and *** denote statistical significance at the 10, 5, and 1% level respectively.

One explanation for our findings are shopper attitudes with regard to masks. While shoppers might be willing to wear a mask while shopping for a shorter period of time for essential goods in grocery stores or other retailers anchoring neighborhood malls, they are less willing to spend an extended period of time leisurely shopping or dining out with masks, which negatively impacts the tenants of regional malls. Overall, our results in Table 3 indicate that our previous findings for state governor (Table 2) can be explained by COVID-19 related restrictions such as mask mandates.

Governmental restrictions in response to COVID-19 are temporary. While impacting the short-term risk perception of investors as captured by the going-in cap rate, they should have a negligible impact on the long-term risk perception. We capture this longer-term risk perception using the pre-tax yield (*PTYLD*) required by respondents to the Situs RERC survey, which is their required return (IRR) for the entire holding period. The average holding period of Situs RERC respondents for regional and neighborhood shopping centers is 10 years, of which the two-year COVID period represents a small fraction. To assess whether our findings for regional malls are indeed driven by shifts in the short-term risk perception due to temporary COVID-19 restrictions, we estimate our model using the pre-tax yield as the dependent variable in our model in Eq. 1.

The results of this robustness check are presented in Table 4. The insignificant coefficient on the interaction effect of *REPUBGOV* and *COVID* suggests that our results in Table 2 were indeed due to the short-term shift in risk perception resulting from temporary COVID-19 restrictions. One explanation for the insignificant coefficient on *REPUBGOV* is that in the long term, i.e., over a 10-year holding period, the party affiliation of governors and policy implications are uncertain and thus do not have an impact on the long-term risk perception of retail investors.

**Table 4:** Robustness Check: Required Pre-Tax Yield

	Regional Mall	
	Coef.	SE
**COVID**	0.39***	**0.08**
**REPUBGOV**	-0.05	0.05
**REPUBGOV#COVID**	-0.06	0.04
**UNEMPL**	-0.02**	**0.01**
**RENPROB**	-0.02*	**0.01**
**MARKT**	0.002	0.02
**INVCOND**	0.05	0.04
**DIVERSE**	0.19***	**0.04**
**POPUL**	< 0.0001	< 0.0001
**POPULGrowth**	-0.03***	**0.01**
**PCI**	-0.00002**	**< 0.0001**
**Constant**	9.15***	**0.81**
*N*	*600*	
*No of groups*	*40*	
*Avg. obs*	*15*	
*Wald χ* ^*2*^	76.80***	

Note: This table presents the results for the regression (panel-specific AR, autocorrelation is calculated based on the autocorrelation of residuals, heteroskedastic panels) for PTYLD. All variables are as defined in Table 1.

*, **, and *** denote statistical significance at the 10, 5, and 1% level respectively.

Our results for the main effect of *REPUBGOV* in Table 2 indicate that the short-term risk perception of retail real estate investors is higher for Republican-led states than for Democratic-led states. However, at the time this investigation was conducted, a number of Republican-led states had Democratic-leaning MSAs such as Miami in Florida, Austin in Texas, or Atlanta in Georgia. To further assess whether our finding for *REPUBGOV* is driven by the political leaning of the overall state or the MSA, we include the political attitudes of an MSA (*REPUBATT*) and its interaction term with *COVID* in our model. Hereby political attitudes of an MSA are measured based on how the respective MSA voted in the 2020 presidential election.^2^*REPUBATT* is coded 1 if the MSA voted Republican, based on the 2020 presidential votes, and 0 otherwise.

The results are shown in Table 5. The coefficients on *COVID* and *REPUBGOV#COVID* for both mall types are in line with our previous findings in Table 2. However, the coefficients on *REPUBGOV* become insignificant for regional and neighborhood malls while the main effect of *REPUBATT* is significantly positive. This suggests that our findings for the main effect, *REPUBGOV*, in Table 2 are driven by the political leanings of MSAs. The risk perception of retail real estate investors is higher for Republican-leaning MSAs than Democratic-leaning MSAs, leading to a higher going-in cap rate requirement. The coefficients on the interaction effect of political attitudes and *COVID* are insignificant for both mall types.

**Table 5:** Results for Going-In Cap Rate, State Governor, and Political Attitudes

	Model 1		Model 2	
	Regional Mall		Neighborhood Center	
	Coef.	SE	Coef.	SE
**COVID**	0.37***	**0.07**	0.24***	**0.06**
**REPUBGOV**	-0.02	0.04	0.05	0.04
**REPUBGOV#COVID**	-0.07***	**0.02**	0.03	0.03
**REPUBATT**	0.26***	**0.07**	0.09**	**0.04**
**REPUBATT#COVID**	-0.01	0.04	-0.01	0.03
**UNEMPL**	-0.01**	**0.006**	-0.01**	**0.005**
**RENPROB**	-0.02*	**0.01**	-0.01**	**0.006**
**MARKT**	0.01	0.02	0.03	0.03
**INVCOND**	0.06*	**0.03**	0.07**	**0.03**
**DIVERSE**	-0.02	0.05	-0.17***	**0.04**
**POPUL**	< 0.0001*	**< 0.0001**	< 0.0001*	**< 0.0001**
**POPULGrowth**	-0.02***	**0.01**	-0.01	0.01
**PCI**	-0.00002***	**< 0.0001**	< 0.0001	< 0.0001
**Constant**	7.77***	**0.76**	6.93***	**0.44**
*N*	*600*		*600*	
*No of groups*	*40*		*40*	
*Avg. obs*	*15*		*15*	
*Wald χ* ^*2*^	118.64***		133.18***	

Note: This table presents the results for the regression (panel-specific AR, autocorrelation is calculated based on the autocorrelation of residuals, heteroskedastic panels) for GICAPR. All variables are as defined in Table 1.

*, **, and *** denote statistical significance at the 10, 5, and 1% level respectively.

One explanation for our findings for *REPUBATT* is that the political attitudes of an MSA proxy for future growth opportunities and thus favorable demand drivers relevant to retailers and retail real estate investors. In our sample, Democratic-leaning MSAs are significantly larger, racially diverse, and have higher incomes than Republican ones. For our sample, households in Democratic-leaning MSAs also spend significantly more on entertainment and apparel & services, based on the BLS Consumer Expenditure Survey. Another explanation relates to the retail real estate asset market. Democratic-leaning MSAs may receive more attention from commercial real estate investors due to the favorable fundamental conditions in the space market, which increases liquidity in the respective asset markets and reduces the risk premium required by investors.

## Conclusion

Political uncertainty and governmental regulations have been found to impact the decision-making of residential and commercial real estate market participants (e.g., Ambrose & Diop, 2021; Luo et al., 2021; Suzuki & Asami, 2020; Asquith, 2019; Diamond et al., 2019a, 2019b; Monfared & Pavlov, 2019; Schuetz et al., 2011; Chau, 1997). However, no previous study has investigated the impact of governmental restrictions on the risk perception of commercial real estate investors. We fill this gap in the literature by using the COVID-19 pandemic as a natural experiment. In particular, we separate the pre-COVID period (2018/2019) from the COVID period (2020/2021), focus on shopping centers, which acknowledges the particularly strong impact of the pandemic on retail real estate (Hoesli & Malle, 2021; Ling et al., 2020; Milcheva, 2022; van Dijk et al., 2020), and proxy for COVID-related governmental restrictions based on the political affiliation of state governors.

Using MSA-level going-in cap rates that reflect ex-ante risk premiums required by investors and thus their risk perception, we find that the COVID-19 pandemic yielded a risk premium compared to the pre-COVID period for neighborhood and regional shopping centers. However, in states with a Republican governor, which proxies for shorter and fewer governmental restrictions, investors required a lower going-in cap rate for regional malls during the COVID-19 period than in Democratic-led states. This provides evidence for the impact of governmental restrictions on the short-term risk perception of investors in a shopping center type significantly affected by mask mandates, social distancing, and store occupancy limitations, which in turn impacted the desire of shoppers to leisurely shop and eat out for extended periods. On the other hand, we find no effect for neighborhood shopping centers, which can be explained with the essential nature of their tenants (e.g., grocery stores).

Further analysis suggests that mask mandates as a type of governmental restriction explain higher risk going-in cap rates, and that pandemic-related governmental restrictions have no impact on the long-term risk perceptions of retail investors. Last, we show that the political leaning of an MSA impacts the risk perception of investors. Explanations for this finding include the signaling effect of political attitudes about factors that impact future space and asset market conditions.

Future studies may use our findings as a starting point to investigate the relation of local political decision-making and commercial real estate investor risk perception. Depending on their political attitudes and interests, decision-makers at commercial real estate development or investment firms may vary in their assessment of risk related to governmental regulations (e.g., rent control, COVID-restrictions) and political climate in a geographical market. Other studies could examine our findings on racial-ethnic diversity in more depth and use them as a starting point for analyzing other dimensions of diversity (e.g., socio-economic status, age, sexual orientation, religious beliefs) and their impact on investor risk perception and commercial real estate market fundamentals. Such investigations could help understand the dynamics underlying our findings on the political attitudes of an MSA and their impact on investor risk perception.

Future investigations can also complement our study by analyzing investor risk perception and behavior for other property types (e.g., office, hotel, multifamily, industrial) in the context of the COVID-19 pandemic. Last, future studies may investigate spillover effects during the pandemic between neighboring states or adjacent MSAs with different levels of governmental restrictions and governor affiliation. These studies could investigate the impact of these spillover effects on the performance of commercial real estate assets and investor attitudes.

## Footnotes

1 For a comprehensive overview into the rights and responsibilities of state governors, please see National Governors Association (NGA): https://www.nga.org/governors/powers-and-authority/.

2 We obtain the data on MSA-level from Richard Florida (Bloomberg, December 4, 2020), based on U.S. Census Bureau data analyzed by Patrick Adler: https://www.bloomberg.com/news/features/2020-12-04/how-metro-areas-voted-in-the-2020-election.

## References

[CR1] Adolph, C., Amano, K., Bang-Jensen, B., Fullman, N., Magistro, B., Reinke, G., Castellano, R., Erickson, M., & Wilkerson, J. (2021). The pandemic policy u-turn: The role of partisanship, public health, and race in decisions to ease COVID-19 social distancing policies in the U.S. *Perspectives on Politics*. 10.7910/DVN/9PFC7P

[CR2] Adolph, C., Amano, K., Bang-Jensen, B., Fullman, N., Magistro, B., Reinke, G., & Wilkerson, J. (2022). Governor partisanship explains the adoption of statewide mask mandates in response to COVID-19. *State Politics & Policy Quarterly*, *22*(1), 24–49. 10.1017/spq.2021.22.

[CR3] Ambrose, B. W., & Diop, M. (2021). Information asymmetry, regulations and equilibrium outcomes: Theory and evidence from the housing rental market. *Real Estate Economics*, *49*(S1), 74–110. 10.1111/1540-6229.12262.

[CR4] Armstrong, J., Skilling, H., & Yao, F. (2019). Loan-to-value ratio restrictions and house prices: Micro evidence from New Zealand. *Journal of Housing Economics*, *44*, 88–98. 10.1016/j.jhe.2019.02.002.

[CR5] Anderson, J. T., Harrison, D. M., & Seiler, M. J. (2022). Reducing Strategic Forbearance under the CARES Act: An experimental Approach utilizing recourse attestation. *Journal of Real Estate Finance and Economics*, *65*, 230–260. 10.1007/s11146-021-09842-4. 38624868 10.1007/s11146-021-09842-4 PMC8120016

[CR6] Asquith, B. J. (2019). Do rent increases reduce the housing supply under rent control? Evidence from evictions in San Francisco. *Upjohn Institute Working Paper*. 10.17848/wp19-296

[CR7] Azzimonti, M. (2018). Partisan conflict and private investment. *Journal of Monetary Economics*, *93*(C), 114–131. 10.1016/j.jmoneco.2017.10.007.

[CR8] Bahmani-Oskooee, M., & Ghodsi, S. H. (2017). Policy uncertainty and house prices in the United States. *Journal of Real Estate Portfolio Management*, *23*(1), 73–85. 10.1080/10835547.2017.12089999.

[CR9] Benton, R. A., Cobb, J. A., & Werner, T. (2021). Firm partisan positioning, polarization, and risk communication: Examining voluntary disclosures on COVID-19. *Strategic Management Journal*, *43*(4), 697–723. 10.1002/smj.3352. 34908629 10.1002/smj.3352 PMC8661910

[CR10] Beracha, E., Freybote, J., & Lin, Z. (2019). The determinants of the ex ante risk premium in commercial real estate. *Journal of Real Estate Research*, *41*(3), 411–442. 10.22300/0896-5803.41.3.411.

[CR11] Bostic, R. W., & Martin, R. W. (2005). Have anti-discrimination housing laws worked? Evidence from trends in black homeownership. *The Journal of Real Estate Finance and Economics*, *31*(1), 5–26. 10.1007/s11146-005-0991-7.

[CR12] Brunner, E., & Sonstelie, J. (2003). Homeowners, property values, and the political economy of the school voucher. *Journal of Urban Economics*, *54*(2), 239–257.

[CR13] Brunner, E., Sonstelie, J., & Thayer, M. (2001). Capitalization and the voucher: An analysis of precinct returns from California’s proposition 174. *Journal of Urban Economics*, *50*(3), 517–536. 10.1006/JUEC.2001.2232.

[CR14] Chan, Y. C., Saffar, W., & Wei, K. J. (2021). How economic policy uncertainty affects the cost of raising equity capital: Evidence from seasoned equity offerings. *Journal of Financial Stability*, *53*, 100841. 10.1016/j.jfs.2020.100841.

[CR15] Chau, K. (1997). Political uncertainty and the Real Estate Risk Premiums in Hong Kong. *Journal of Real Estate Research*, *13*(3), 297–315. 10.1080/10835547.1997.12090878.

[CR16] Chetty, R., & Hendren, N. (2018). The impacts of neighborhoods on intergenerational mobility II: County-level estimates. *The Quarterly Journal of Economics*, *133*(3), 1163–1228. 10.1093/qje/qjy006.

[CR17] Chetty, R., Hendren, N., Kline, P., Saez, E., & Turner, N. (2014). Is the United States still a land of opportunity? Recent trends in intergenerational mobility. *American Economic Association*, *104*(5), 141–147. 10.3386/w19844.

[CR18] Chin, M. K., Hambrick, D. C., & Treviño, L. K. (2013). Political Ideologies of CEOs. *Administrative Science Quarterly*, *58*(2), 197–232. 10.1177/0001839213486984.

[CR19] Choudhry, T. (2020). Economic policy uncertainty and house prices: Evidence from geographical regions of England and Wales. *Real Estate Economics*, *48*(2), 504–529. 10.1111/1540-6229.12266.

[CR20] Clayton, J., Ling, D., & Naranjo, A. (2009). Commercial real estate valuation: Fundamentals versus investor sentiment. *Journal of Real Estate Finance and Economics*, *38*, 5–37. 10.2139/ssrn.1132361.

[CR21] Çolak, G., Durnev, A., & Qian, Y. (2017). Political uncertainty and ipo activity: Evidence from U.S. gubernatorial elections. *Journal of Financial and Quantitative Analysis*, *52*(6), 2523–2564. 10.1017/S0022109017000862.

[CR22] Cortright, J. (2018). *Identifying America’s Most Diverse, Mixed Income Neighborhoods*. Portland, OR: City Observatory. Retrieved from http://cityobservatory.org/lost-in-place/.

[CR23] D’Lima, W., Lopez, L. A., & Pradhan, A. (2021). COVID-19 and housing market effects: Evidence from U.S. shutdown orders. *Real Estate Economics*, *50*(2), 303–339. 10.1111/1540-6229.12368.

[CR24] Dehring, C. A., Depken, C., & Ward, M. (2008). A direct test of the homevoter hypothesis. *Journal of Urban Economics*, *64*(1), 155–170.

[CR25] Deng, X., Anglin, P. M., Gao, Y., & Sun, H. (2021). How do the ceo political leanings affect REIT business decisions? *Journal of Real Estate Research*, *43*(4), 419–446. 10.1080/08965803.2021.2003507.

[CR26] Di Giuli, A., & Kostovetsky, L. (2014). Are red or blue companies more likely to go green? Politics and corporate social responsibility. *Journal of Financial Economics*, *111*(1), 158–180. 10.1016/j.jfineco.2013.10.002.

[CR27] Diamond, R., McQuade, T., & Qian, F. (2019a). The effects of rent control expansion on tenants, landlords, and inequality: Evidence from San Francisco. *American Economic Review*, *109*(9), 3365–3394. 10.1257/aer.20181289.

[CR28] Diamond, R., McQuade, T., & Qian, F. (2019b). Who pays for rent control? Heterogeneous landlord response to San Francisco’s rent control expansion. *AEA Papers and Proceedings*, 109, 377–380. 10.1257/pandp.20191021

[CR29] Diaz, J., Hansz, A., Cypher, M., & Hayunga, D. (2008). Conservation status and residential transaction prices: Initial evidence from Dallas, Texas. *Journal of Real Estate Research*, *30*(2), 225–248. 10.1080/10835547.2008.12091216.

[CR30] Duanmu, J., Li, Y., Lin, M., & Tahsin, S. (2022). Natural disaster risk and residential mortgage lending standards. *Journal of Real Estate Research*, *40*(1), 106–130. 10.1080/08965803.2021.2013613.

[CR31] Feng, Z., & Wu, Z. (2021). Local economy, asset location and REIT firm growth. *The Journal of Real Estate Finance and Economics*. 10.1007/s11146-021-09822-8.

[CR32] Fisher, G., Steiner, E., Titman, S., & Viswanathan, A. (2022). Location density, systematic risk, and cap rates: Evidence from REITs. *Real Estate Economics*, *50*(2), 366–400. 10.1111/1540-6229.12367.

[CR33] Francis, B. B., Hasan, I., & Zhu, Y. (2014). Political uncertainty and bank loan contracting. *Journal of Empirical Finance*, *29*(C), 281–286. 10.1016/j.jempfin.2014.08.004.

[CR34] Frey, W. H. (2021). *2020 Census: Big cities grew and became more diverse, especially among their youth*. Retrieved from: https://www.brookings.edu/research/2020-census-big-cities-grew-and-became-more-diverse-especially-among-their-youth/#:~:text=Big%20cities%20became%20even%20more,growth%20in%20the%20past%20decade.

[CR35] Gulen, H., & Ion, M. (2015). Policy uncertainty and corporate investment. *Review of Financial Studies*, *29*(3), 523–564. 10.1093/rfs/hhv050.

[CR36] Gupta, A., Fung, A., & Murphy, C. (2021). Out of character: CEO political ideology, peer influence, and adoption of CSR executive position by Fortune 500 firms. *Strategic management journal*, *42*(3), 529–557. 10.1002/smj.3240.

[CR37] Hoechle, D. (2007). Robust standard errors for panel regressions with cross-sectional dependence. *The Stata Journal*, *7*(3), 281–312. 10.1177/1536867X0700700301.

[CR38] Hoesli, M., & Malle, R. (2021). Commercial real estate prices and COVID-19. *Journal of European Real Estate Research*. 10.1108/JERER-04-2021-0024.

[CR39] Hoyos, R. (2006). Testing for cross-sectional dependence in panel-data models. *Stata Journal*. 10.1177/1536867X0600600403. 6SarafidisV.

[CR40] Hoyt, W. H., Coomes, P. A., & Biehl, A. M. (2011). Tax limits and housing markets: Some evidence at the state level. *Real Estate Economics*, *39*(1), 97–132. 10.1177/1536867X0600600403.

[CR41] Hutton, I., Jiang, D., & Kumar, A. (2014). Corporate policies of republican managers. *Journal of Financial and Quantitative Analysis*, *49*(5), 1279–1310. 10.1017/S0022109014000702.

[CR42] Jens, C. E. (2017). Political uncertainty and investment: Causal evidence from U.S. gubernatorial elections. *Journal of Financial Economics*, *124*(3), 563–579. 10.1016/j.jfineco.2016.01.034.

[CR43] Jeong, N., & Kim, N. (2020). The effects of political orientation on corporate social (ir)responsibility. *Management Decision*, *58*(2), 255–266. 10.1108/MD-06-2019-0713.

[CR44] Julio, B., & Yook, Y. (2012). Political uncertainty and corporate investment cycles. *The Journal of Finance*, *67*(1), 45–83. 10.1111/j.1540-6261.2011.01707.x.

[CR45] Kaiser, C., & Freybote, J. (2021). Is e-commerce an investment risk priced by retail real estate investors? An investigation. *Journal of Property Research*, *39*(3), 197–214. 10.1080/09599916.2021.1996447.

[CR46] Liao, W. C., Luo, Y., & Sun, Y. (2022). Information shock of disaster and hazard: Impact of Kaohsiung gas explosions and risk disclosure on the equalizing difference in the housing market. *Real Estate Economics*. 10.1111/1540-6229.12382.

[CR47] Lima, R. C. A., & Neto, S., R.d.M (2019). Zoning ordinances and the housing market in developing countries: Evidence from brazilian municipalities. *Journal of Housing Economics*, 46. 10.1016/j.jhe.2019.101653.

[CR48] Ling, D. C., Wang, C., & Zhou, T. (2020). A first look at the impact of COVID-19 on commercial real estate prices: Asset-level evidence. *The Review of Asset Pricing Studies*, *10*(4), 669–704. 10.1093/rapstu/raaa014.

[CR49] Luo, S., Tidwell, A., & Clements, S. (2021). Does political uncertainty affect residential development? *The Journal of Real Estate Finance and Economics*. 10.1007/s11146-021-09867-9.

[CR50] Milcheva, S. (2022). Volatility and the cross-section of real estate equity returns during Covid-19. *Journal of Real Estate Finance and Economics*, *65*, 293–320. 10.1007/s11146-021-09840-6. 38624822 10.1007/s11146-021-09840-6 PMC8087339

[CR51] Monfared, S., & Pavlov, A. (2019). Political risk affects real estate markets. *The Journal of Real Estate Finance and Economics*, *58*(1), 1–20. 10.1007/s11146-017-9619-y.

[CR52] Nguyen, N. H., & Phan, H. V. (2017). Policy uncertainty and mergers and acquisitions. *Journal of Financial and Quantitative Analysis*, *52*(2), 613–644. 10.1017/S0022109017000175.

[CR53] Pence, K. (2022). Liquidity in the mortgage market: How does the COVID-19 crisis compare with the global financial crisis? *Real Estate Economics*, *50*(6), 1405–1424. 10.1111/1540-6229.12389.

[CR54] Pew Research Center (2020). *Republicans and Democrats move even further apart in coronavirus concerns*. Retrieved from: https://www.pewresearch.org/politics/2020/06/25/republicans-democrats-move-even-further-apart-in-coronavirus-concerns/.

[CR55] Pew Research Center (2021). *Where Democrats and Republican align and differ on COVID-19 issue*. Retrieved from: https://www.pewresearch.org/fact-tank/2021/03/24/despite-wide-partisan-gaps-in-views-of-many-aspects-of-the-pandemic-some-common-ground-exists/.

[CR56] Pollack, A. B., & Kaufmann, R. K. (2022). Increasing storm risk, structural defense, and house prices in the Florida Keys. *Ecological Economics*, *194*, 107350. 10.1016/j.ecolecon.2022.107350.

[CR57] Schuetz, J., Meltzer, R., & Been, V. (2011). Silver bullet or trojan horse? The effects of inclusionary zoning on local housing markets in the United States. *Urban Studies*, *48*(2), 297–329. 10.1177/0042098009360683. 21275196 10.1177/0042098009360683

[CR58] Subrahmanyam, V., Singh, M., & Pennathur, A. (2020). CEO characteristics, firm performance, and corporate political contributions. *Review of Financial Economics*, *38*(2), 379–404. 10.1002/rfe.1082.

[CR59] Suzuki, M., & Asami, Y. (2020). Tenant protection, temporal vacancy and frequent reconstruction in the rental housing market. *Real Estate Economics*, *48*(4), 1074–1095. 10.1111/1540-6229.12205.

[CR60] Takeda, Y., Kono, T., & Zhang, Y. (2019). Welfare effects of floor area ratio regulation on landowners and residents with different levels of income. *Journal of Housing Economics*, *46*, 101656. 10.1016/j.jhe.2019.101656.

[CR61] van Dijk, D., Kinsella Thompson, A., & Geltner, D. (2020). Recent drops in market liquidity may foreshadow major drops in us commerical real estate markets. *SSRN Electronic Journal*. 10.2139/ssrn.3604606.

[CR62] Waisman, M., Ye, P., & Zhu, Y. (2015). The effect of political uncertainty on the cost of corporate debt. *Journal of Financial Stability*, *16*(C), 106–117. 10.1016/j.jfs.2015.01.002.

[CR63] Wang, C., & Zhou, T. (2022). Face-to-Face Interactions, Tenant Resilience, and Commercial Real Estate Performance. *Real Estate Economics*, forthcoming, 10.1111/1540-6229.12412

[CR64] Xu, M., & Xu, Y. (2020). Environmental hazards and mortgage credit risk: Evidence from Texas pipeline incidents. *Real Estate Economics*, *48*(4), 1096–1135. 10.1111/1540-6229.12213.

[CR65] Yi, D., & Choi, H. (2020). Housing market response to new flood risk information and the impact on poor tenant. *The Journal of Real Estate Finance and Economics*, *61*(1), 55–79. 10.1007/s11146-019-09704-0.

[CR66] Zahirovic-Herbert, V., & Turnbull, G. K. (2009). Public school reform, expectations, and capitalization: What signals quality to homebuyers? *Southern Economic Journal*, *75*(4), 1094–1113.

[CR67] Zhang, L., Leonard, T., & Bitzan, J. (2022). Impacts of the COVID-19 Pandemic on House Prices: Heterogeneous Impacts over Time and across the House Price Distribution. *Journal of Real Estate Research*, forthcoming, 10.1080/08965803.2022.2041272

[CR68] Zhang, W., & Logan, J. R. (2016). Global neighborhoods: Beyond the multiethnic metropolis. *Demography*, *53*(6), 1933–1953. 10.1007/s13524-016-0516-4. 27778294 10.1007/s13524-016-0516-4 PMC5513176

[CR69] Zhou, Z., Li, H., & Zhang, A. (2022). Does Bike Sharing increase House Prices? Evidence from Micro-level Data and the Impact of COVID-19. *Journal of Real Estate Finance and Economics*, forthcoming, 10.1007/s11146-022-09889-x 10.1007/s11146-022-09889-x PMC8817944 40479230

